# Regulating Ruminative Web Browsing Based on the Counterbalance Modeling Approach

**DOI:** 10.3389/frai.2022.741610

**Published:** 2022-02-11

**Authors:** Junya Morita, Thanakit Pitakchokchai, Giri Basanta Raj, Yusuke Yamamoto, Hiroyasu Yuhashi, Teppei Koguchi

**Affiliations:** ^1^Department of Behavior Informatics, Faculty of Informatics, Shizuoka University, Hamamatsu, Japan; ^2^Graduate School of Integrated Science and Technology, Shizuoka University, Hamamatsu, Japan; ^3^Department of Socio-Information Studies, Faculty of Informatics, Shizuoka University, Hamamatsu, Japan

**Keywords:** ACT-R, cognitive modeling, web advertisement, internet addiction, heart rate, nudge, mental health, homeostasis

## Abstract

Even though the web environment facilitates our daily life, emotional problems caused by its incompatibility with human cognition are becoming increasingly serious. To alleviate negative emotions during web use, we developed a browser extension that presents memorized product images to users in the form of web advertisements. This system utilizes the cognitive architecture Adaptive Control of Thought-Rational (ACT-R) as a model of human memory and emotion. A heart rate sensor attached to the user modulates the ACT-R model parameters, and the emotional states represented by the model are synchronized (following the chameleon effect) or counterbalanced (following the homeostasis regulation) with the physiological state of the user. An experiment demonstrates that the counterbalanced model suppresses negative ruminative web browsing. The authors claim that this approach, utilizing a cognitive model, is advantageous in terms of explainability.

## 1. Introduction

Even though the information provided by the web has improved a broad range of factors in our lives and in society, several emotional problems, which were previously rare, have emerged. Issues such as repetitive internet use and anxiety during internet searches are becoming increasingly serious, occasionally inducing negative social collective behaviors, such as flaming, cyberbullying, and cyberstalking.

Many prominent examples can be found during the early stage of the worldwide COVID-19 pandemic. Especially in countries like Japan, there were several cases of amplification of negative emotion toward infected persons through social network services (SNS) (e.g., Jinnai, 2020; Nomura, 2020). In such cases, SNS users with anxious feelings fragmentarily found the victims' personal information, then their feelings expanded by encountering similar attitudes toward the victims. Finally, uncontrollable collective blame was directed toward the victims. Although some cultural factors might be involved in these examples, we can find similar cases globally. In fact, social and legal systems for regulating such issues have been actively discussed (e.g., General Data Protection Regulation, 2018; Jones, 2018). However, interventions based on the understanding of the underlying cognitive and emotional mechanisms remain underdeveloped.

Concerning these mechanisms, it has been pointed out that the negative side effects of technology, which are as severe as drug addiction, become emphasized and lead to repetitive overuse (Alter, 2017). In this discussion, it was claimed that digital technology makes behavioral addiction serious because it enables users to obtain information effortlessly. Other authors have also pointed out that behavioral addiction induced by digital technology is particularly problematic if users have mental health problems (Twenge et al., 2018). Among these problems, this study focuses on rumination, which is a psychological state related to a depressive mood and involves repetitive negative thinking on a specific topic. Technologically enhanced and socially accumulated ruminative thinking may have a severe impact on society, along with the synergistic action of the echo-chamber effect, which amplifies beliefs or attitudes through interactions among a large number of individuals (Del Vicario et al., 2016; Wollebæk et al., 2019).

In this study, we propose an approach of implicit behavioral prompting (a type of nudge proposed by Thaler and Sunstein, 2009) based on cognitive models (computational representations of the internal processes underlying the mind (e.g., Fum et al., 2007; Stewart and Myers, 2021) to prevent such negative behavior during web browsing. The proposed approach also utilizes monitoring the the users' physiological states. Specifically, we propose a counterbalance modeling approach that maintains an internal affective state following the principle of homeostatic regulation (Cannon, 1929; Billman, 2020). In this approach, a cognitive model presents information to the user to keep a physiological baseline state. This paper presents and tests this approach using the following organization: First, we review related work on the problem and the approach. Subsequently, we describe the system of model-based advertisements and experimentally evaluate it using a mood-induction procedure. In the final section, we discuss implications including ethical issues and future directions.

## 2. Related Studies

Herein, we present a detailed background for this study: (a) a problem statement, (b) user modeling, and (c) behavior change techniques.

### 2.1. Ruminative Thinking and Behavioral Addiction

This study is concerned with rumination, which is commonly defined as repetitive and negative thinking about unpleasant experiences such as disappointments or past mistakes (Nolen-Hoeksema and Morrow, 1991; Treynor et al., 2003). Rumination is considered a serious mental health issue, and it has been claimed to be a preceding stage of depression. As is the case with other depressive symptoms, rumination prolongs dysphoric moods and causes attentional biases toward negative information (Mogg et al., 1995; Cramer et al., 2016), that is, ruminating people are attracted to negative information more easily, resulting in worsened depressive symptoms. The level of severity varies depending on the individual. In some cases, people who often ruminate face a greater risk of depression (Kuehner and Weber, 1999).

Combining such a negative mental state with information technology sometimes causes a more severe feedback loop accompanied by negative feelings. Information technology removes the limits from cognitive boundaries that have evolved throughout the history of humanity. Humans naturally forget information that is not relevant to their current situation (Anderson and Schooler, 1991; Schacter, 2002). However, the web can instantly provide information that does not decay with time. This convenience induces addictive behavior, and people cannot resist using digital technology even when they are aware of the irrationality of their own behavior (Alter, 2017).

### 2.2. Cognitive Modeling and Cognitive Architecture

Considering that emotional problems related to the web are caused by incompatibility with human memory, we focus on a user model that coordinates between natural and artificial cognitive systems. Among several approaches to modeling human cognition, the Adaptive Control Thought-Rational [ACT-R: Anderson, 2007] was selected in this study. The ACT-R is one in a series of successively developed cognitive architectures [see Kotseruba and Tsotsos (2018) for an exhaustive review]. In this context, the term “architecture” indicates a modeling framework consisting of structured modules. Each module in a cognitive architecture is assigned with a primary mental function, such as vision, goal management, data storing, or rule execution. Utilizing these modules makes it possible to construct a cognitive model that simulates mental processes that occur during a specific task. Among several existing cognitive architectures, the ACT-R architecture is suitable for building a model of the memory process during web browsing because it has a *declarative module*. This module functions as a database with peculiar features that simulate the retrieval process of human declarative memory (i.e., specific episodes or general semantic knowledge).

In fact, the theorization of declarative memory occupies a central position in ACT-R; it refers to the activation mechanism for each memory item, which affects the likelihood that the item will be retrieved successfully and quickly. The calculation of activation is largely controlled by the frequency and recency of each individual memory (Anderson and Milson, 1989). Frequently or recently retrieved memory items have high activation. In addition, memory decay and the spacing effect impact activation. The forgetting curve theory (Ebbinghaus, 1885) is concerned with memory decay, resulting in loss of information over time if this information is not recalled. In contrast, according to the spacing effect theory (Ebbinghaus, 2013), frequently recalling information may strengthen its retention, that is, a piece of information becomes more difficult to forget after being periodically recalled^1^.

Following such a memory activation mechanism, ACT-R naturally produces ruminative behavior; repetitive recalls of past experiences raises the priority of a small number of memories, leading to a continuous cycle of retrieving specific memories. In fact, Lebiere and Best (2009) pointed out that a free recall made by the normal ACT-R model leads to “*pathological behaviors such as out-of-control looping*.” Moreover, during rumination, the brain continues retrieving memories based on their priority; nonetheless, the negative experiences that cause rumination are the most likely to be recalled. Recent research has explored the simulation of such negative memory retrieval using ACT-R. Van Vugt et al. (2015) constructed an ACT-R model that simulates the processes of mind-wandering. In this model, a state of attention on a task is suppressed by falling into the mind-wandering state in which the model continuously recalls past memories until it is reminded to return to its task. In another study (Van Vugt et al., 2018), the previous ACT-R model was applied with moods (cheerful, content, down, suspicious, and insecure) in place of memories, and a model that simulates ruminating participants was implemented. The model supports the assumption that the activation of negative mood leads to the cyclic retrieval of highly prioritized memory, showing consistency with an empirical study (Van Vugt et al., 2012).

In the present study, we apply the above findings regarding the mechanism of human memory to a system regulating ruminative behavior during web browsing. Besides the simulation studies exploring the nature of human cognitive processes, the ACT-R has been used as user models to support human activity in a series of studies. A well-known example can be found in education. Since its early success, the concept of an *intelligent tutoring system* (ITS) has been greatly developed (Anderson et al., 1985). However, the approach of ITS is only applicable to fields where the required knowledge is well-defined, such as mathematics, science, or programming. In contrast to this traditional approach, recent studies on ACT-R modeling have increasingly included affective processes (Ritter, 2009; Dancy et al., 2015; Juvina et al., 2018). Based on such models, several methods of emotional support have been proposed. For example, Morita et al. (2016) developed a method called *model-based reminiscence* in which the model contains user lifelog data as declarative memory, and presents these memory data in accordance with the retrieval mechanism implemented in ACT-R. This framework has been expanded by Itabashi et al. (2020) to include physiological data such as heart rate so that the retrieval parameter may be modulated in real time. However, model-based reminiscence is limited because the system cannot intervene in ruminative web browsing during the activity.

### 2.3. Prompting Behavior Change

To support activity during web browsing, we should develop an information presentation method that does not strongly intervene in the main tasks. The concept of a *nudge* refers to an approach realizing such an implicit intervention that mildly changes individual behaviors and decisions (Thaler and Sunstein, 2009). This concept itself includes various approaches, as reviewed by Caraban et al. (2019). Among the techniques categorized in the human-computer interaction (HCI) field, implicit intervention in human cognition has been found to have great potential to reinforce behaviors through drawing attention at appropriate times. For example, Zhu et al. (2017) studied the effects of implicit prompts on encouraging computer users to correct their sitting posture. Ibragimova et al. (2015) designed a smart driving system that collects data from an accelerometer to detect driver behavior and provides feedback through LED lights and vibrations on the steering wheel, nudging drivers to drive less aggressively. Summarizing these studies, it is conceivable that nudge techniques (mild implicit prompts for behavior change) could be extended to address mental issues, particularly rumination, and be applied to prevent users from continuing rumination during web browsing.

## 3. Model-based Advertisements

**Figure 1:**
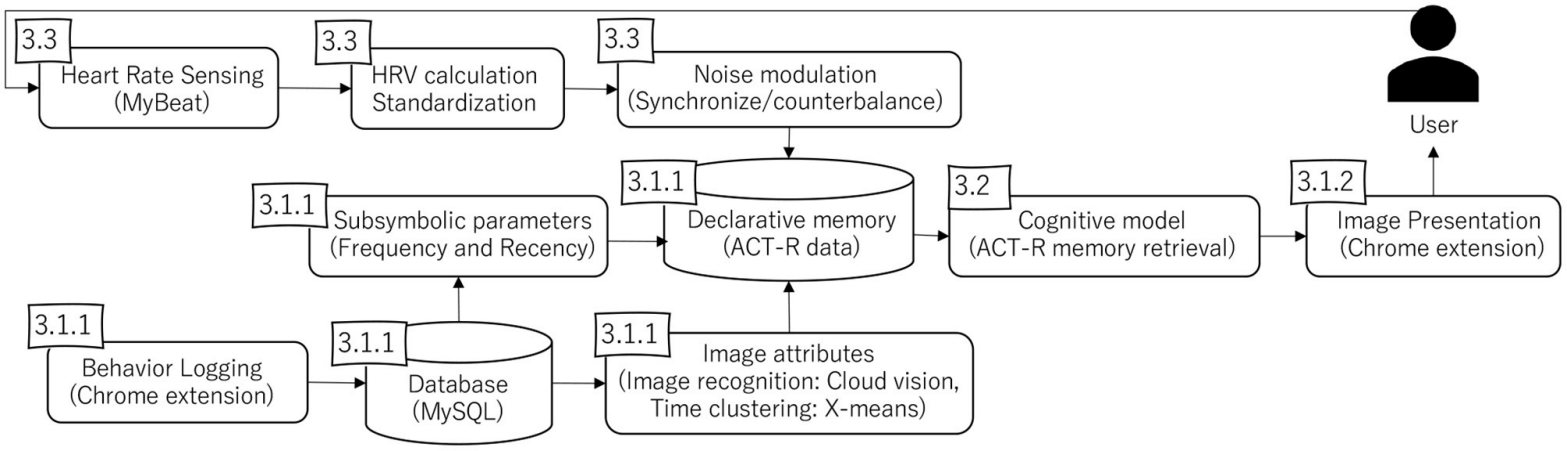
System overview. The numbers in the boxes indicate the sections describing each component. The data stored by the behavior logging (3.1.1) is transformed into data compatible with the ACT-R cognitive model (3.2), which outputs sequential image presentations (3.1.2). The user's reaction is monitored by heart rate sensing (3.3), to make the model synchronize or counterbalance with the user's state.

The proposed system extends the memory model developed in previous studies (Morita et al., 2016; Itabashi et al., 2020) to regulate ruminative behavior during web browsing. To naturally apply the memory model in a web environment, we focused on web advertisement such as behavioral targeting. Several studies have indicated the potential of behavioral changes toward healthy behavior through this type of online media (Kramer et al., 2014; Yom-Tov et al., 2018). In our system, the visited product images are always presented in the right region of a web page. The images are periodically changed to affect the implicit memory processes of the user. Figure 1 shows an overview of the system. In the rest of this section, we explain the implementation of the system, which comprises three parts: (a) browser extensions, (b) a cognitive model, and (c) heart rate sensing. The Supplementary Material in this paper also includes an example of the actual behavior of the system as a movie.

### 3.1. Browser Extensions

The system is implemented in a web environment based on the widely used web browser Google Chrome. To customize the browsing experience on Chrome, Google provides a tool to develop “extensions” built on web technologies (such as HTML, CSS, and JavaScript). Using this tool, we developed the following two independent extensions:

#### 3.1.1. Behavior Logging

This extension collects the user's experienced events in natural web browsing activities. When the user visits specific shopping sites, such as Amazon^2^ and Rakuten^3^, the extension automatically collects the images (source URLs) displayed on the browser screen and stores them in a database (MySQL)^4^. on a server. The stored URLs are automatically converted into data according to the ACT-R declarative memory. In this process, Google cloud vision (cloud-based image recognition software)^5^ assigns labels to the images, and the visited timestamp is also digitized by *X*-means clustering (Pelleg et al., 2000). These attributes (labels and time periods) connect the observed images, forming a network that allows the model to retrieve images according to their semantic association successively (Morita et al., 2016).

In the ACT-R framework, these attributes are represented as *symbolic chunks*, referring to discrete information units. In contrast, ACT-R has *subsymbolic parameters*, referring to numeric values attached to symbolic chunks. In the current study, each image is assigned with two subsymbolic parameters: the number of times shown on the browser and the time since each presentation. These parameters are respectively used to simulate human memory's frequency and recency effects (Anderson and Milson, 1989) as described later.

#### 3.1.2. Image Presentation

The images stored in the database are retrieved by another Chrome extension that is connected with the ACT-R model. When this extension is enabled by the user, the model returns an image URL that is converted into an image by the browser. The ACT-R model and the Chrome extension communicate using TCP/IP, and the image in the right region of the browser is refreshed every 5 s according to the output of the model^6^.

This refresh interval follows Morita et al. (2016) and Itabashi et al. (2020), where the ACT-R memory model was used in a photograph presentation system. Regarding this setting, it should be noted that conventional advertisements in digital media set longer intervals than those used by our model. For example, according to Constantin et al. (2018), advertisements in mobile apps are refreshed at 30–60 s or greater, aiming to improve economic values mainly represented by the click-through rate. In contrast, our system seeks to affect the implicit memory process instead of increasing clicking behaviors. Moreover, a finding indicates that fast animation on web banners elicits physiological arousal and high memory recall rates about the contents (Sundar and Kalyanaraman, 2004). Considering these factors, this study adopted the same refresh interval as the previous studies (Morita et al., 2016; Itabashi et al., 2020), although it was quite short for advertising a single product.

### 3.2. Cognitive Models

In addition to the refresh rate, we followed the above previous studies regarding image retrieval mechanisms; we adopted the same ACT-R model used by Itabashi et al. (2020) except that the present model employs product images rather than private photographs. Thus, the model has declarative memory representing visited product images, and their retrieval is regulated using the standard activation equation of ACT-R 6.0 (Bothell, 2007).


(1) $$\begin{array}{l} A_{i}=B_{i}+S_{i}+\epsilon_{i} \end{array}$$

The activation value (*A*_*i*_) of memory item *i* is calculated as the sum of the base-level activation (*B*_*i*_), the strength of association (*S*_*i*_)^7^, and probabilistic noise (ε_*i*_). Among these factors, the base-level activation is crucial for representing the ruminative behavior, and it is calculated as follows:


(2) $$\begin{array}{l} B_{i}=\ln\left(\overset{n}{\underset{j\;=\;1}{\sum }}t_{j}^{-d}\right)+\beta_{i} \end{array}$$

where *n* is the number of occurrences of memory item *i*, *t*_*j*_ is the time elapsed since the *j*th occurrence, *d* is the decay factor, and β_*i*_ is the offset value. As noted in 3.1.1, the present study extracted the values of *n* and *t* from the user's everyday web browsing activities, whereas the other parameters were fixed at default values according to ACT-R (*d* = 0.05, β = 0). Recently collected and frequently visited product images receive high activation after being applied with this equation and the setting.

With the base-level activation alone, the ACT-R memory naturally converges to a specific memory item, leading to repetitive displays on the browser as already noted in 2.2. One solution to escaping this repetitive memory recall is to modulate the noise parameter ε_*i*_ which was generated using a Gaussian distribution of mean 0, and variance


(3) $$\begin{array}{l} \sigma =\left(\pi /3\right)\times s^{2} \end{array}$$

where *s* is a parameter that determines the size of the noise variance. If the value of ε_*i*_ fluctuates widely, the possibility of escaping the feedback loop increases (Morita et al., 2016).

Regarding the mechanism of fluctuating such a noise level, Ritter (2009) proposed representing emotion as a modulator of subsymbolic parameters in cognitive processes. A more specific theory was proposed by Dancy et al. (2015). Their theory, called ACT-R/ϕ (pronounced *act-are-phi*), extends ACT-R to include physiological processes to represent the influence of emotion on cognitive function. The key concept of this theory is connecting the subsymbolic parameters of ACT-R to physiological dynamics. In particular, they adopt an integrated physiological simulator called HumMod (Hester et al., 2011) to predict fluctuations of the peripheral nervous system (PNS) caused by stressful stimuli and the passing of time. Such PNS fluctuations are tied to subsymbolic parameters of ACT-R. Among several assumed relations, they especially emphasized the connection between the aforementioned noise variance [*s* in Equation (3)] and the activation of noradrenaline, which related to anxiety and high arousal level (Mizuki et al., 1996). Although they did not reach a conclusion over a single definitive function connecting these two variables, several alternatives were tested in a serial subtraction task.

Based on the theory by Dancy et al. (2015), Itabashi et al. (2020) developed a real-time parameter modulation method targeted at the current user. In this method, the noise parameter is replaced with the physiological state of the user, which is directly obtained from a heart rate sensor. Connecting ACT-R's subsymbolic parameter to the user's physiological state in real-time, Itabashi et al. (2020) aimed to realize synchronous interaction between the system and the user. Thus, if the user's heart rate indicates a relaxed state, the model retrieves images according to a parameter connected with a low arousal state. In contrast, if the user's heart rate shows a stressful state, the model's parameter is adjusted to represent a high arousal state. Itabashi et al. (2020) assumed that such synchronous interaction is adequate to provide a comfortable feeling to the user because of the chameleon effect, which refers to the idea that mimicking the other increases positive impressions of social interaction (Chartrand and Bargh, 1999).

The present study follows the idea of real-time modulation presented by Itabashi et al. (2020), but a new connection method is tested in addition to the synchronous method described above. In the following subsection, both methods are described in detail.

### 3.3. Heart Rate Sensing

The system collects physiological data from a heart rate monitor and converts them into the noise parameter of ACT-R. Specifically, the R-R interval (RRI), which indicates an interval between successive heartbeats, is constantly collected through a wearable heart rate monitor (myBeat WHS-1, Union Tool Co. Japan), which is attached onto the chest of the user and is connected with the server through Bluetooth. While the ACT-R model is running on the server, the program converts the data to *s* in Equation (3) in real time. This procedure follows the previous study by Itabashi et al. (2020) and consists of the following three steps:

1. Heart rate variability calculation:
  The heart rate monitor collects and sends the RRI every three beats in the form of a single signal. The program first calculates the heart rate variability (HRV) from the standard deviation of the three most recent RRIs according to the following equation:
(4) $$\begin{array}{l} HRV_{i}=\sqrt{\left(\sum {\left(x_{i}-\mu \right)}^{2}\right)/n} \end{array}$$
  where *x* is a single RRI. Although several variations represent this measure, HRV generally reflects the degree of relaxation (oppositely stress). It is well known that fluctuations in RRI increase in the parasympathetic dominance state (Schaaff and Adam, 2013; Nardelli et al., 2015).
2. Standardization:
  The standard score of the HRV is calculated by comparing it with the mean of the baseline data (μ) collected when the user is in a relaxed state:
(5) $$\begin{array}{l} z=\left(HRV_{i}-\mu_{b}\right)/SD_{b} \end{array}$$
  where *i* represents the latest data, and *b* represents the baseline data. Thus, this equation standardizes each HRV based on the variance obtained in a baseline session.
3. Conversion of HRV to the noise parameters:
  Finally, the standard score is converted to the parameter *s* by adding an offset value of 0.5 and setting it to zero if it is below zero:
(6) $$s=\{\begin{array}{ll} z+0.5, & \text{if }z+0.5>0\\ 0, & \text{otherwise} \end{array}$$

The parameter *s* as calculated by the above procedure leads to a large fluctuation in memory retrieval when the user is relaxed (parasympathetic dominance state). On the other hand, when the user feels stressed and anxious (sympathetic dominance state), the activation value calculated by Equations (1) and (2) almost always outputs the same images, having a pronounced recency and frequency effect. This behavior by the model is compatible with psycho-physiological studies showing lower HRV during worry and ruminative thinking (Brosschot et al., 2006). Therefore, the model by Itabashi et al. (2020), where *s* corresponds to the HRV, synchronizes ruminative behavior with the participants.

**Figure 2:**
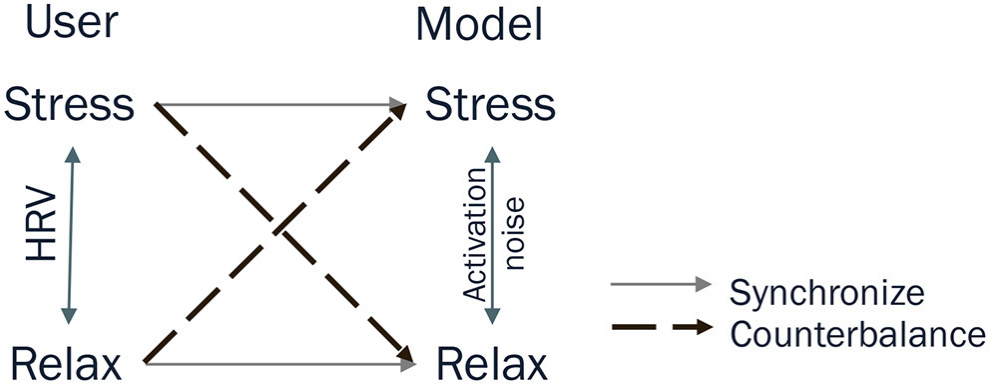
Connection between a user and a cognitive model. The states of the user (left) and the model (model) are located along the vertical axes indicating the arousal level. High and low arousal levels correspond to stressed and relaxed situations, respectively. The heart rate variability (HRV) and the activation noise [*s* in Equation (3)] represent the axis of the user and the model. The synchronize relation (horizontal arrows) directly connects these two variables, whereas the counterbalance relation (diagonal arrows) inverts HRV [Equation (7)] before connecting with the activation noise.

Considering the above, the synchronous interaction seems unable to improve the user's mental health problem even if the assumed chameleon effect exists. Instead, there is a possibility of worsening the problem because the mechanism is similar to an echo-chamber (Del Vicario et al., 2016; Wollebæk et al., 2019). Therefore, unlike the previous study, this study hypothesizes that the counterbalance model in which *s* is the inverse of the HRV can aid in suppressing ruminative behavior. Figure 2 shows the schematic representation of the model-user relation in the previous study (synchronous) and the present study (counterbalance). Accordingly, we modified the calculation. Specifically, after Equation (4), we inserted an intermediate step by inverting the HRV and multiplying by the average of the baseline as follows:


(7) $$\begin{array}{l} HRV_{inv}=HRV_{i}^{-1}\times \mu_{b} \end{array}$$

Using this *HRV*_*inv*_, the modified model proceeds with the above steps 2 and 3. In this process, the baseline data of HRVs are also inverted. As a result, this model presents various images to the user when s/he ruminates, whereas it repeats the same image when the user is in a relaxed state. Such a counterbalance relation follows the principle of homeostatic regulation in a healthy normal human (Cannon, 1929; Billman, 2020) to maintain a physiological baseline state. Therefore, it can be assumed that the model showing counterbalance behavior regulates mental health problems (ruminative behavior) by diverting their attention from internal thinking to various product images.

## 4. Experiment

An experiment was conducted to test the efficacy of the proposed system in terms of suppressing ruminative behavior. Achieving this outcome requires inclining participants to ruminate. Therefore, we created a session to adjust the mood of the participants using mood induction procedures (MIPs), which are psychological methods that influence the emotions of the participants prior to the experiment (Westermann et al., 1996). MIP techniques are largely divided into two types based on the mood sources external (e.g., movies or music) and internal sources (e.g., imagination or memory). Among them, a type of MIPs called *autobiographical recollections* (Brewer et al., 1980; Schwarz and Clore, 1983), classified into the latter source, seems to be suitable for the current study. It asks participants to recall past experiences that evoke a specific mood. For the aim of the current study, negative memories recalled by this method were assumed to lead to ruminative thinking. Under this assumption, we investigated whether the participants, with the aid of the proposed system, could recover from ruminative web browsing.

### 4.1. Method

#### 4.1.1. Participants

The experiment was conducted from June to July 2020 under the approval of the Ethical Committee of Shizuoka University. Due to practical reasons described later, this experiment could not collect a large sample size. Under the condition that the experiment could be executed during the period, we recruited 12 Japanese participants (six males and six females) who reported that they often visit shopping websites (Amazon or Rakuten) through personal contact. All of them were undergraduate or graduate students majoring in informatics. They received a reward of 1,500 JPY for their participation.

#### 4.1.2. Design

The participants were randomly divided into two groups (each comprising six people): synchronized (sync) and counterbalanced (coun). For the former, we used the ACT-R model with the *s* parameter corresponding to the HRV [Equation (4)], whereas for the latter, we used the ACT-R model with *s* being the inverse of the HRV [Equation (7)]. As noted earlier, the model used in the counterbalance condition presents diverting images when the participant ruminates. Such image presentations are considered to be effective in regulating ruminative thinking. Therefore, we hypothesized that the coun group would outperform the sync group in suppressing ruminative web browsing.

#### 4.1.3. Questionnaire

To evaluate the effectiveness of the proposed system, the participants were asked four questions:

1. “*How much did you recognize the images shown on the right region of the screen?”*
2. “*How much did you find interesting the images?”*
3. “*How much did you remember negative events from your memory?”*
4. “*How annoying did you find the images when the images were changed?”*

Each question corresponds to an aspect presented in Table 1. These aspects broadly correspond to steps that a person goes through when changing their behavior following receptions of external prompts. Especially, the three aspects (1, 2, 4) were adopted from a previous study that evaluated explicit impressions of a prompt interface aimed at behavioral changes (Zhu et al., 2017). Contrary, the third item (distraction) was originally employed according to the aim of the study. The third aspect directly evaluates the suppressing effect of the system on negative memory induced by a MIP described below. In the present study, all of the aspects were rated on a seven-point Likert-scale (1–7: very little to very much), except for the third aspect, for which a lower score indicates that the participants were distracted from negative memory retrieval to a larger degree. Examining the participants' responses to these questions, we will discuss the subjective effect of the system both as general prompts toward behavior change and prompts specific to suppressing ruminative thinking.

**Table 1:** Aspects of post-experiment questions.

**Aspect**	**Description**
1) Recognition	The extent to which the participant can recognize the images.
2) Attention	The extent to which the participant is interested in the images.
3) Distraction	The extent to which the images can distract the participant from negative memory retrieval.
4) Annoyance	The extent to which the images annoy the participant.

#### 4.1.4. Procedure

The procedure involved the following steps:

1. Behavior logging:
  The participants downloaded and installed the extension for behavior logging on the computer they usually used. The duration from the download to the next step of the experiment was three to five weeks. During this period, the participants were asked to browse products on shopping websites.
2. Instructions regarding the tasks:
  The objective and the entire procedure were explained to the participants. The aim was described as “*examining the influence of memory recall by a system based on a cognitive model processing the user's web browsing history.”* Then, they were informed that the experiment included three tasks: “*In the first and the second task, you will be asked to recall a recent memory and collect information about your future life, respectively. In the final task, you will write a report summarizing these two tasks.”* The final task was not actually carried out, but this instruction was made to maintain the influence of the mood induced by the first task on the second task. Although the relation between the two tasks was not directly mentioned to the participants, they needed to retain their recalled memory to write the final report during the second task. We considered that this setting is suited to induce ruminative behavior in the second task.
  After the above instructions were provided and the participants agreed to participate in the experiment, they were asked to sign a consent form. They were also told that they could leave the experiment anytime if they felt pain during the procedure.
3. Baseline measurement:
  Following the instructions, the participants attached the heart rate monitor to their chest. After it was confirmed that the heart-beat data were transmitted correctly, the baseline session was initiated by letting all participants relax for approximately 3 min. The system utilized 50 HRVs (15–64th) obtained in this session to calculate the baseline used in Equation (5).
4. Mood induction task:
  In this task, the participants recalled an unpleasant memory that occurred within the past six months and was most frequently recalled in recent times. This condition of autobiographical recollection follows Schwarz and Clore (1983)'s experiment. As in this previous study, we also asked participants to remind the memory “*as vividly and in as deeper as possible*.” It could be any negative story that frequently came into their mind. More specifically, we explained that the negative events in this procedure might relate to the feeling of regret, embarrassment, and suffering. Within a time limit of 15 min, they listed the events and wrote them down those in a Microsoft Word document. They were told that the written document would be used as materials in the third task. Therefore, they did not need to use whole sentences in this step.
5. Main task:
  In the main task, the participants were asked to assume that “*they are going to start a new working life anywhere you want next spring.”* They were told to spend 15 min browsing websites and searching for what they thought would be necessary for realizing their plans. The content of the choices of the participants could be interpreted broadly. We assumed that participants who were unable to stop recalling negative experiences would be likely to ruminate in this type of open-ended question. During the task, the image presentation system operated to display product images selected by the ACT-R model. Although it was not explicitly stated to the participants, we assumed that the product images collected in the behavior logging period could provide cues to gather information about goods required in future life.
6. Questionnaire:
  After the time was up, we explained that the summary task was a dummy. We finished the experiment by having the participants complete the post-experiment questionnaire presented in Table 1.

### 4.2. Results

The data obtained in the experiment were analyzed to study the effect of the counterbalance modeling approach on the recovery process from mood induction. Owing to the limited number of participants, non-parametric statistical methods were adopted. Specifically, we used the Wilcoxon rank-sum test to examine the difference between the two groups. To compensate for the high Type II error rate, we set the significance level to *p* < 0.10. The statistical power (1-β) calculated by the *post-hoc* power analysis for detecting large effect size (*d* = 0.8) at alpha level (*p* < 0.10) under the above condition (*n* = 12) is 0.318.

#### 4.2.1. Model Behaviors

**Figure 3:**
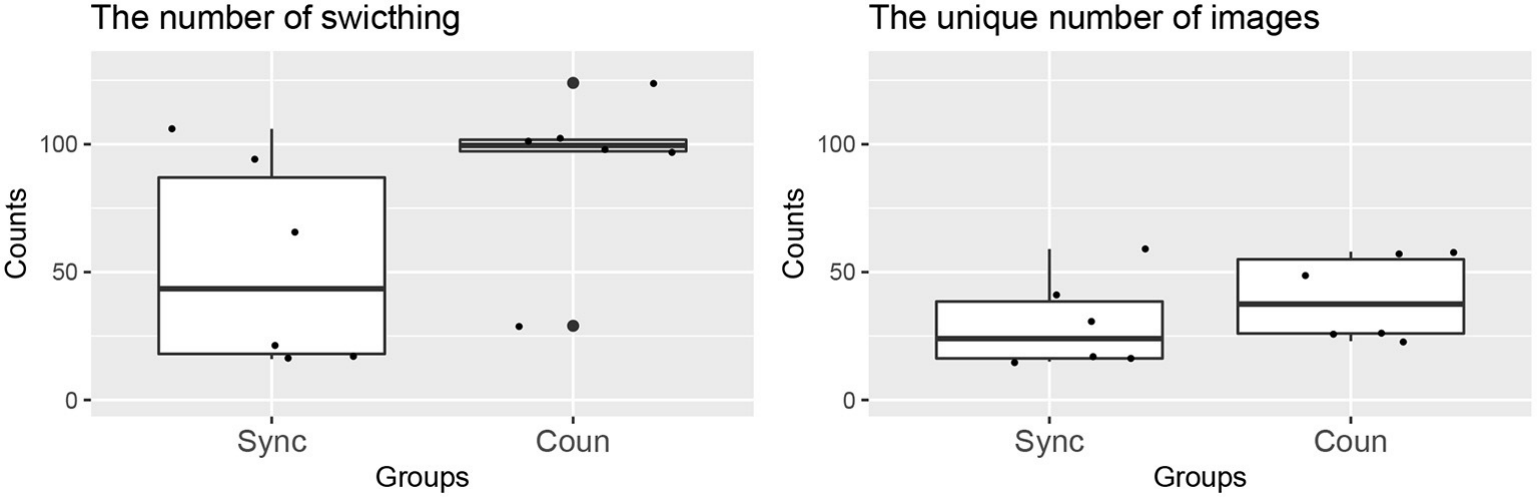
Counting results for model behavior.

As explained previously, the system refreshes the browser images every 5 s. However, owing to the recency and frequency effect introduced by Equation (2), the same images tend to be shown on the display. Therefore, we examined the effect of the counterbalance model by counting the total number of image switches and the unique number of images, excluding duplicates. The counts are shown in Figure 3, where a significant difference in the total number of switches between the two groups can be observed (*p* = 0.09). In contrast, the difference in the unique number of images is not significant (*p* = 0.37). These results indicate that the two models appear to behave differently although the difference is not obvious in these simple indices.

#### 4.2.2. Questionnaire Answers

**Figure 4:**
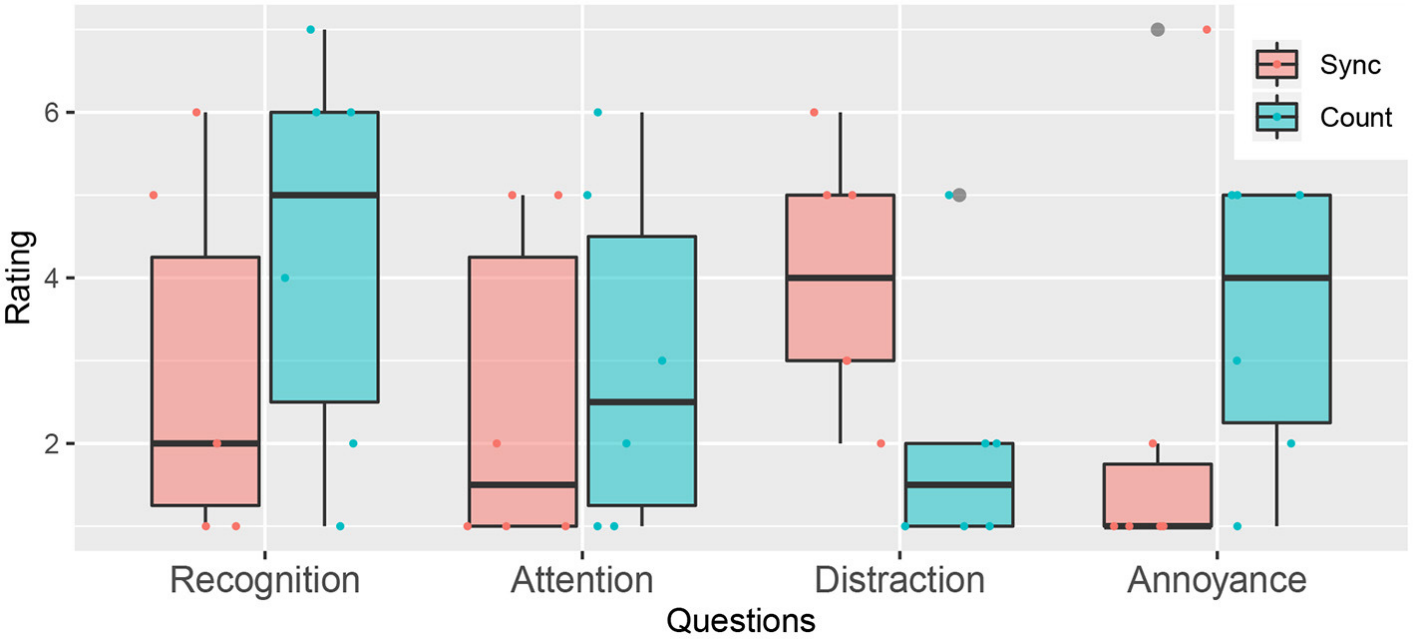
Evaluation scores for the post-experiment questions.

To study the subjective effect of the system, we compared the scores obtained in the questionnaire, as shown in Figure 4. Among the four questions, a significant difference was found regarding distraction (*p* = 0.04). Accordingly, the effect of the proposed approach on suppressing negative memory retrieval was confirmed. Contrary, no significant difference between the two groups was observed regarding the other aspects (recognition: *p* = 0.65; attention: *p* = 0.65; annoyance *p* = 0.180). From these, we consider that the system has a subjective effect on suppressing ruminative thinking, whereas the subjective effect on prompting general behavior change is not so explicit.

#### 4.2.3. Heart Rate Data

**Figure 5:**
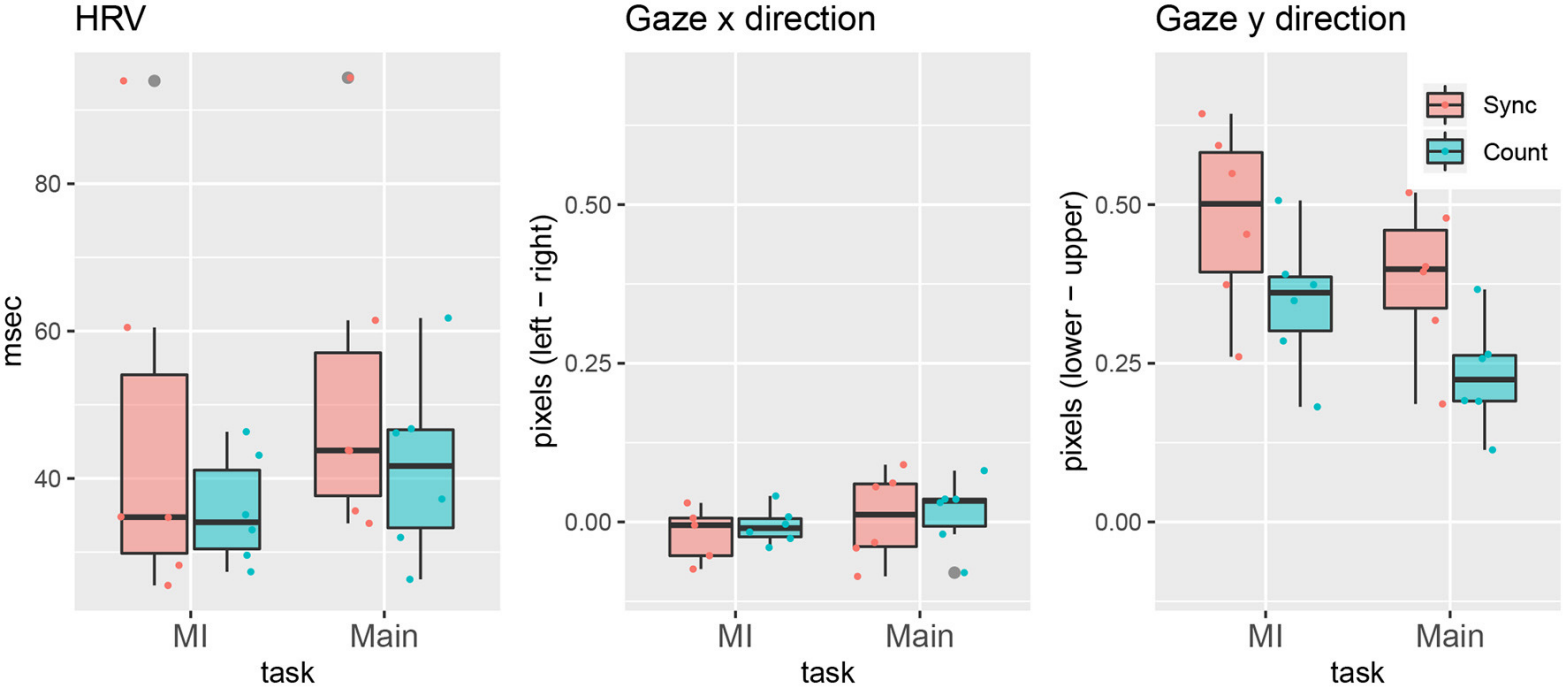
HRV and gaze distributions.

**Figure 6:**
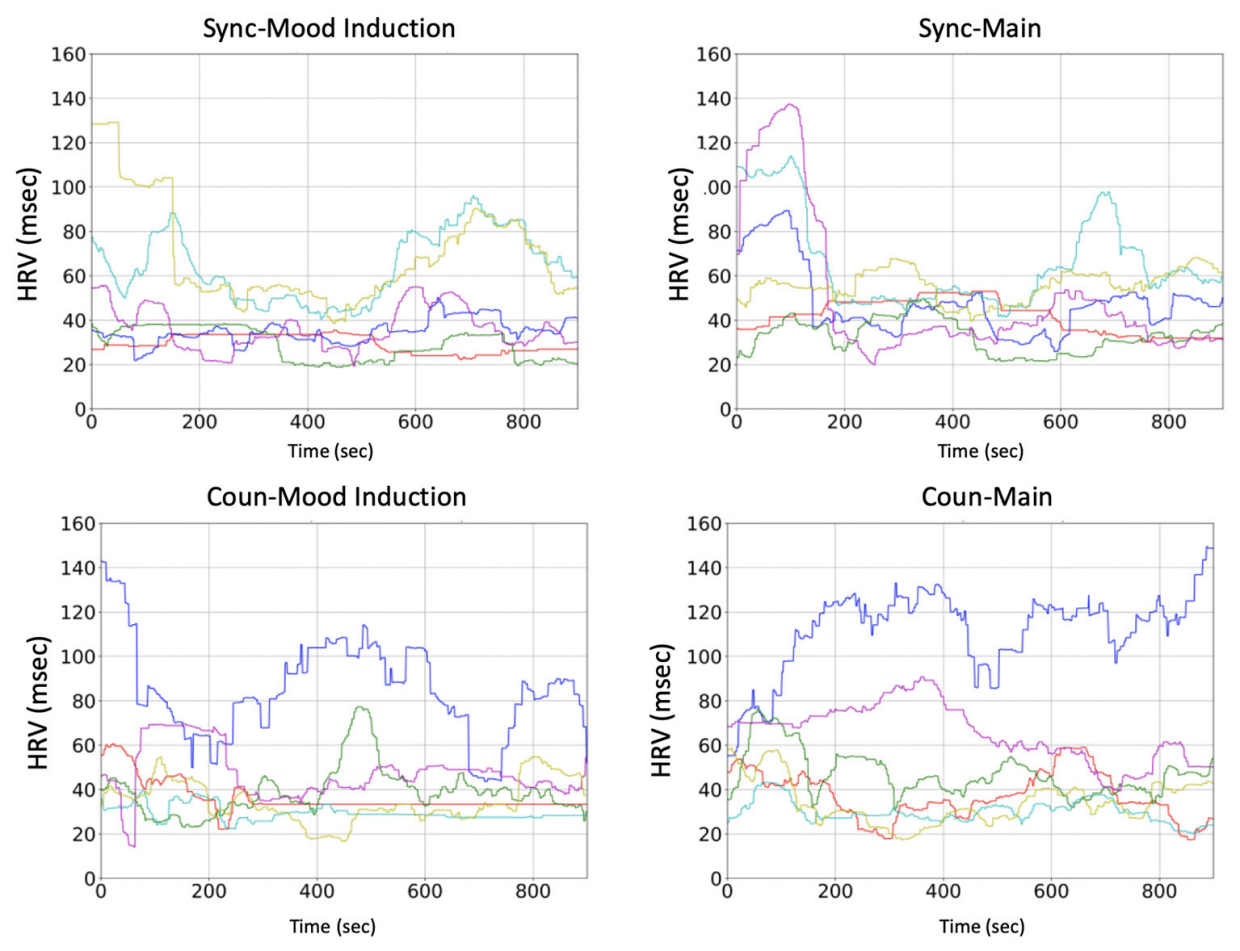
Fluctuations of HRV during the two tasks (**left:** mood induction; **right:** main task). Each line indicates a participant in the two groups (**top:** sync; **bottom:** coun).

The graph on the left of Figure 5 shows the boxplots of the HRV calculated by Equation (4), which were summarized from Figure 6 and indicate the fluctuations in HRV between the two tasks. The boxplot indicates the differences between the two tasks in both groups: The HRV in the mood-induction task was smaller than that in the main task. This result may have occurred because the negative memory induced by the mood induction task might lead to a small HRV or the participants were nervous at the start of the experiment. For whatever reason, in the main task, the participants recovered their mood while browsing for their future life.

Unlike the difference between the two tasks, the difference between the two groups in terms of the recovering effect was not obvious. We tested the difference between the groups by comparing the difference of the HRV from the main task to the mood induction task. No significant difference between the groups was observed regarding this index (median in sync: 7.90, median in coun: 2.26, *p* = 0.81).

#### 4.2.4. Gaze Information

We examined the difference in gaze information during the tasks. As the interface used in this study presents images on the screen, we assumed that the influence of the system appeared in the gaze movement. In this analysis, we used the OpenFace software package (Baltrusaitis et al., 2018) to extract gaze information from a video recorded during the tasks. In the experiment, the faces of the participants were recorded with a web camera (1080p, 30 fps) set on the top of the display. Among several indices output by OpenFace, we used the *x* and *y* values of the vectors directed from the eyes on the image captured by the camera.

**Figure 7:**
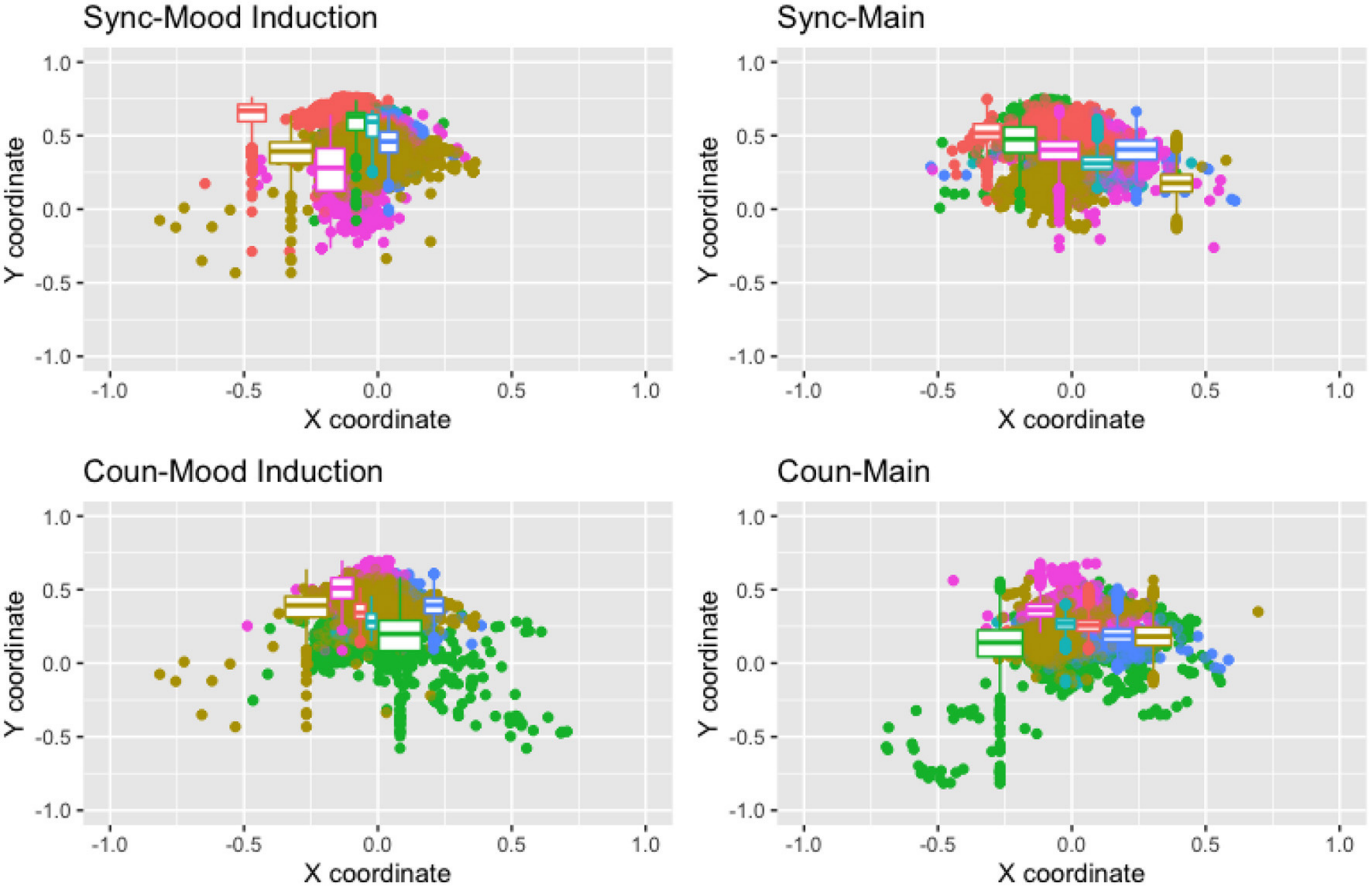
Gaze distribution in the two tasks (**left:** mood induction; **right:** main task). The colors identify the participants in the two groups (**top:** sync; **bottom:** coun).

Figure 7 shows the gaze distribution of the participants for each task in each group, presenting the *x*-*y* coordinates as dots obtained each second (averaging 30 frames per second). The dot color distinguishes the participants, and the boxplots in the figure indicate summaries of the dot distribution for each participant. Although there were large individual differences between the participants in terms of this measure, we can find a difference between the tasks in both groups. To clarify this difference, we further summarized the gaze distribution for each participant, as shown in the two panels on the right of Figure 5, where each dot corresponds to the averaged *x*-*y* coordinates for each participant. In the main task, the vectors from the eyes were directed rightward lower than in the mood induction task, suggesting the participants mainly observed the left part of the screen (the contents of the web page). However, the difference between the two groups in terms of this tendency is less clear. Again, we calculated the difference from the main task to the mood-induction for each *x* and *y* coordinate. No significant difference was found for either the horizontal (median in sync: 0.04, median in coun: 0.03; *p* = 0.58) or the vertical direction (median in sync: 0.11, median in coun: 0.11; *p* = 1.00) in this index.

#### 4.2.5. Correlation Analysis

So far, a significant difference between the two groups has been found only in the evaluation of distraction. To confirm the reliability of this difference, we calculated Spearman's rank correlation coefficients between the distraction rating and the other indices. Table 2 summarizes the results of this calculation for each group. A significant correlation in the number of switches in the counterbalance model (*p* = 0.03) can be observed. This result suggests that the increase in switching by the counterbalance model is correlated with high negative memory retrieval. The horizontal gaze direction also showed significant correlations with distracting negative memory retrieval in the sync (*p* = 0.05) and coun groups (*p* = 0.09), although the direction of the correlation was different. In the sync group, attention toward the web contents (the left screen) is correlated with distraction from negative memory retrieval, whereas the attention toward the web contents in the coun group is correlated with the negative memory retrieval. In other words, in the coun group, the distraction by the web contents is correlated with the distraction from the negative memory retrieval, suggesting that web advertisements had an effect.

**Table 2:** Spearman's correlation coefficient between the distraction and other indices.

	**Sync**	**Coun**
Recognition	−0.060	−0.547
Attention	0.127	−0.046
Annoyance	−0.069	−0.557
The total number of switches	−0.117	0.833^*^
The unique number of images	−0.029	−0.172
Difference of x-gaze from main to MI	−0.794^+^	0.740^+^
Difference of y-gaze from main to MI	−0.588	−0.617
Difference of HRV from main to MI	−0.088	−0.308

+ *p < 0.10*,

* *p < 0.05*.

## 5. Discussion and Conclusion

### 5.1. Summary and Implications

To regulate ruminative web browsing, we developed a system that consists of a cognitive model of memory using ACT-R, physiological sensing to modulate memory retrieval, and image presentations implemented as a browser extension. This combination is considered to follow the principles of affective computing (Picard, 1995, 2003), which emphasizes the integration of computational models of affect and emotion, emotion recognition using multi-modal data, and emotion expression affecting the mental state of users.

The contribution of the present study is to extend the principle of affective computing by including a computational cognitive modeling of memory. This model differs from the previous behavioral model (Fogg, 2009) used in web advertisement (Yom-Tov et al., 2018) in that it includes internal memory processes. Although there are several options for modeling emotion and memory [e.g., Friston (2010); Schmidhuber (2010), as mathematical models of curiosity], we claim that including the ACT-R cognitive architecture provides another theoretical basis of implicit-prompting systems designed to adapt the emotional states of users based on an academic field with a long history.

Furthermore, we empirically confirmed the effect of model-based prompts in the condition where the model maintains user homeostasis. The effect of this counterbalance approach was demonstrated in the subjective evaluation. The correlation analysis also validated this evaluation, indicating correlations with the number of switches and the gaze directions toward the advertisements. These correlations confirmed that the proposed mechanism is effective in distracting negative memory retrieval. Thus, we can summarize the experiment by observing the recovery process in subsequent web-searching: the effect of the approach was observed in terms of users forgetting their negative memories. Accordingly, the proposed approach can be seen as a possible candidate for achieving harmony between natural and artificial cognitive systems, balancing emotional issues in this digital age.

### 5.2. Limitations and Future Works

Despite its advantages, the proposed approach has several limitations that should be addressed in the future. Specifically, there is a lack of strong evidence for the effectiveness of the counterbalance model in terms of behavioral and physiological indices. The statistical tests in Figure 5 could not indicate significant effects demonstrating the advantages of the counterbalance approach. As can be seen in Figures 6, 7, the variance from individual differences is so large that the number of participants (statistical power) should be increased.

Regarding the limited number of participants, there are two reasons in the present study. One of the reasons is the high cost of the experiment. This experiment requires a long preparation period, and it collects a large amount of physiological data. The other reason is that the experiment was conducted amidst the worldwide COVID-19 pandemic, where human activities were spontaneously suppressed. Concerning the latter reason, we consider that the experiment conducted in such a situation, where a high level of anxiety is expected, is also worth reporting, although the small number of participants is a limitation we should overcome in the future.

Another limitation is the experimental setting under which only the negative mood was induced. In such a situation, the model in the counterbalance condition frequently changes images to distract attention from negative memories. However, the users' moods in the real-world setting are complex and diverse. Therefore, to fully utilize the counterbalance modeling approach, testing in different experimental settings is needed. These settings include when the user's mood is too optimistic to concentrate on a specific task. By inducing such an incredibly relaxed mood, users' HRV will become high, and the counterbalance model will present fewer images. It is worth to address whether there is a benefit of the proposed approach in such a situation.

The limitation of the above experimental settings also questions the need for adaptive modulation using HRV. Some readers may find it sufficient to simply present images frequently without a parameter modulation by HRV. However, concerning this question, the present study alone can claim that the frequent changes of image without monitoring the user's state is insufficient to suppress negative memory retrieval. The correlation analysis presented in Table 2 indicates non-monotonic relations between the model behavior and the suppression of negative memory retrieval. Focusing on the coun group, we observe that frequent switching rather promoted negative memory retrieval. This non-monotonicity suggests the advantage of adaptive modulation to maintain an optimal level of arousal (Yerkes and Dodson, 1908). In a future study, we will further explore methods of setting such an optimal level of arousal.

Finally, in a future study, we should analyze the relation between the contents of the reminded memory in the mood induction task and searching behaviors in the main task. Although this research targeted ruminative behavior, we have not presented the occurrence of such behavior, so far. Regarding the written text in the mood induction task, Table 3 summarizes the contents of the texts classified by the category grouping frequently observed topics. From the table, we could not find a significant difference between the two conditions^8^. Concerning the searching history in the main task, some participants appeared to reflect the nervousness induced by the respective memory. For example, a participant who noted regret regarding his/her decision to attend graduate school also performed a search related to financial issues in the lives of graduate students in the main task. Another participant also used search keywords related to the remote work environment, reflecting the pandemic situation at the time. However, it was difficult to confirm the severity of such ruminative behavior from the existing data alone. In a future study, we will develop an analysis method to quantify ruminative behavior during web browsing.

**Table 3:** Classification of the written texts in the mood induction task.

	**Sync**	**Coun**	**Example**
After graduation (Job hunting / Graduate school)	2	2	When I was job hunting, I was unable to answer unexpected questions during an interview with a career advisor.
COVID-19	4	1	Due to the influence of the coronavirus, I couldn't find a part-time job, and it was hard to realize that I was annoying my parents.
Death of a friend	1	1	I went to visit the grave of my classmate when I was in high school.
Death of pet animal	1	1	The cat I kept at my parents' house has died.
Game (addiction)	1	2	I was annoyed to lose in the game. I was frustrated by what I was not good at. I was angry that I was taken time to lose and not have fun.
Human relation	1	2	Organization management of club activities. All 6 people cannot work together.
Research / School works	4	3	I had to use programming, but I wasn't good at it and I had to learn by myself, so it was hard when I was thinking about programming.

*The categories were post-determined from the collected text. The columns named sync and coun indicate the numbers of the participants who wrote texts classified into the corresponding categories. The examples were arbitrarily extracted from the written texts and translated from Japanese*.

### 5.3. Ethical Stance

As a final remark in this paper, the ethical aspects of this study should be stated. The study was conducted to prevent ruminative web browsing based on the belief that this personal behavior relates to social problems. If most people in cyberspace could regulate their behavior at will, many social disputes (e.g., Jones, 2018) would not occur. However, implicit prompts such as advertisements have always been a problem, as one can control the emotions of others without their consent. In this regard, the cognitive model-based approach may be a better choice because cognitive modeling has explicit parameters with shared consensus in academic communities, and, therefore, explainability is higher than in machine-learning user modeling.

We also believe that the methods of parameter modulation such as counterbalancing or synchronizing in this study should be selected by users. Even people with severe depression should be allowed to select how their behavior should be regulated. There is a moment when they can manage to make future plans (Kornfield et al., 2020). Therefore, we believe that open and clear discussion made in academic communities will eventually overcome the problem caused by the technology developed in the community itself.

## Data Availability Statement

The raw data supporting the conclusions of this article will be made available by the authors, without undue reservation.

## Ethics Statement

The studies involving human participants were reviewed and approved by Shizuoka University Research Ethics Committee. The patients/participants provided their written informed consent to participate in this study.

## Author Contributions

JM, YY, HY, and TK contributed to conception and design of the study. TP conducted the system implementation and conduct the experiment. GR extracted the gaze information from the video data. JM visualizes the data and conducted the statistical tests. TP and JM wrote the draft of the manuscript. All authors contributed to manuscript revision, read, and approved the submitted version.

## Funding

This study was conducted under the Topic-Setting Program to Advance Cutting-Edge Humanities and Social Sciences Research administrated by Japan Society for the Promotion of Science.

## Conflict of Interest

The authors declare that the research was conducted in the absence of any commercial or financial relationships that could be construed as a potential conflict of interest.

## Publisher's Note

All claims expressed in this article are solely those of the authors and do not necessarily represent those of their affiliated organizations, or those of the publisher, the editors and the reviewers. Any product that may be evaluated in this article, or claim that may be made by its manufacturer, is not guaranteed or endorsed by the publisher.

## Footnotes

^1^The theory of memory in ACT-R is heavily influenced by caching algorithms such as the Least Recently/Frequently Used (LRFU) policies (Lee et al., 1996). Christian and Griffiths (2016) introduced an interview with J. R. Anderson, saying that he developed a theory of human memory by referring to the literature of information retrieval.

^2^ https://www.amazon.com/

^3^ https://www.rakuten.com/

^4^ https://www.mysql.com/

^5^ https://cloud.google.com/vision

^6^This refresh interval is the minimum time to change the image on the screen. If the model's output is the same as the previous one, the screen will not change.

^7^This paper does not go into detail of this parameter because it does not directly relate to ruminative thinking.

^8^We conducted Fisher's exact test for the largest difference (COVID 19) and could not find a significant difference (*p* = 0.24).
